# Congenital intrahepatic aorto-portal fistula presenting with cardiac failure

**DOI:** 10.1259/bjrcr.20200006

**Published:** 2020-07-10

**Authors:** Adithya Pathanki, Khalid Sharif, Ian McCafferty, Jane Hartley, Simon McGuirk

**Affiliations:** 1 The liver unit, including small bowel transplantation, Birmingham Children’s Hospital, Steelhouse lane, Birmingham, United Kingdom; 2 Department of Interventional Radiology, Queen Elizabeth Hospital Birmingham, Mindelsohn way, Birmingham, United Kingdom; 3 Department of Interventional Radiology, Birmingham Children’s Hospital, Steelhouse lane, Birmingham, United Kingdom

## Abstract

Congenital intrahepatic arterio-portal fistulae (cIAPF) are rare, high-flow vascular malformations that usually present with portal hypertension. They almost never cause heart failure, unless there is associated congenital heart disease or the ductus venosus in patent.

We present an unusual case of IAPF in an 11-day-old boy, who presented with features of cardiac failure associated with increased N-terminal pro-brain natriuretic peptide (NT pro-BNP). The IAPF arose directly from the aorta, separated from the hepatic artery and divided to separately supply both left and right portal veins. The ductus venosus was occluded. The IAPF was treated with embolization of the aorto-portal fistula, accessed through a direct percutaneous puncture of the fistula. Embolization was associated with an immediate clinical improvement and a rapid and sustained normalization of the NT pro-BNP level. A similar re-presentation was noted and treated with repeat embolization. The child is well on follow-up.

To our knowledge, this is the first case of cIAPF, which was presented with cardiac failure when the ductus venosus has closed and has been treated successfully with direct, percutaneous transhepatic embolization of the fistula, twice. Serial clinical follow-up and ultrasonographical examinations have proven to be an effective strategy to detect recurrent fistulae.

## Introduction

Congenital intrahepatic arterio-portal fistulae (cIAPF) are rare, high-flow hepatic vascular malformations.^1^ In the adult population, arterio-portal fistulae are known to occur in the setting of trauma or as a complication of interventions to the liver such as ablations. cIAPF are characterized by an intrahepatic communication between the systemic arterial and portal venous systems without any communication with the systemic venous system, and present before 18 years of age.^2^ cIAPF usually present in infancy or early childhood with symptoms of portal hypertension, including gastrointestinal bleeding, ascites and malabsorption.^1,2^ By contrast, they almost never present with heart failure.^3^ We report an unusual case of a direct aorto-portal fistula, presenting with congestive cardiac failure, which was managed by direct puncture of the fistula and percutaneous embolization, twice.

## Case report

An 11-day-old Caucasian male was referred for evaluation of an apparent cardiac murmur associated with poor feeding, tachypnoea during feeds and failure to thrive since birth. The cardiovascular examination was normal and an echocardiogram demonstrated a structurally normal heart. However, examination revealed respiratory distress with tachycardia, tachypnoea, mild intercostal recession and hepatomegaly, with a liver edge palpable 3 cm below the costal margin, together with a prominent systolic liver murmur.

There were no other points in the child’s presentation and family history that could point to an established diagnosis like hereditary haemorrhagic telangiectasia.

The child had been delivered by emergency caesarean section for poor intrauterine growth and decreased foetal movements. However, his initial postnatal course had been unremarkable. In particular, umbilical catheterization had not been performed and there was no history of abdominal trauma or previous surgical intervention.

Haematological and liver function tests were normal. However, the N-terminal pro-brain natriuretic peptide (NT pro-BNP) was increased (2816 ng ml^−1^; normal <85 ng ml^−1^). This measurement, together with the clinical presentation, was kept with a diagnosis of congestive cardiac failure. He was commenced on diuretic therapy which provided symptomatic relief and high calorie nasogastric feeding to supplement his growth.

**Figure 1.**
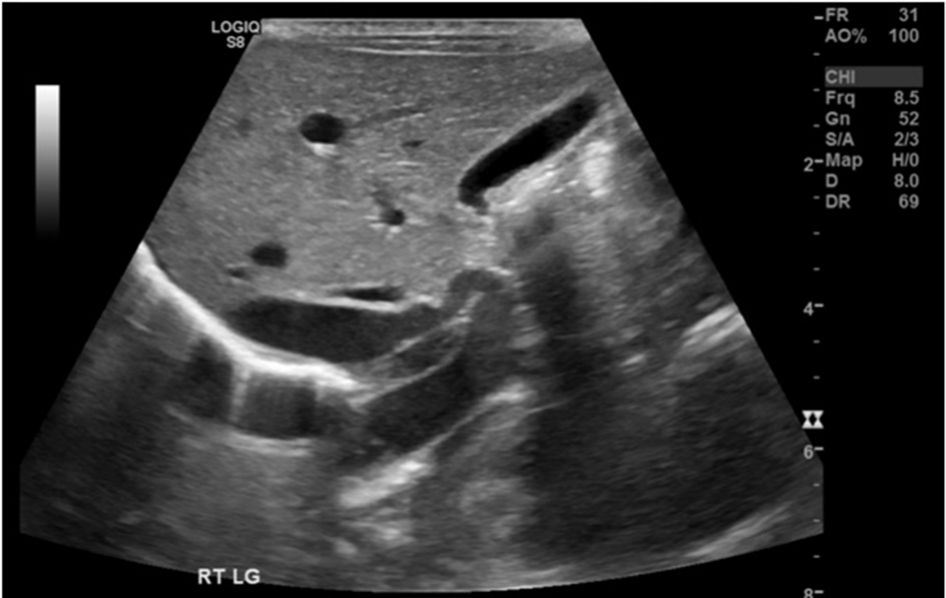
Primary diagnostic ultrasound revealing cIAPF.

Abdominal ultrasonography demonstrated features in keeping with a cIAPF. There was a large, tortuous arterial vessel arising from the aorta that communicated via an intrahepatic fistula with the portal venous system (Figure 1). There was pulsatile, hepatofugal flow in the main portal vein. The inferior vena cava and hepatic veins appeared normal.

**Figure 2.**
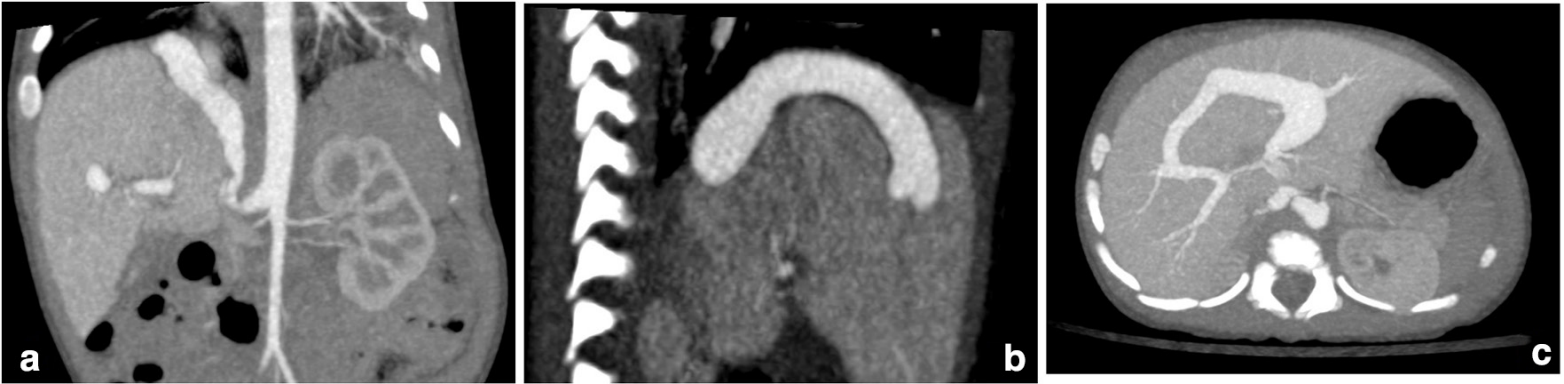
Primary CT scan showing cIAPF in coronal (a), saggital (b) and axial (c) views.

Dual-phase contrast-enhanced CT scan was performed to clarify the diagnosis and plan treatment. This demonstrated a large arterial vessel, which arose directly from the abdominal aorta immediately superior to the level of the coeliac axis (Figure 2). This vessel formed a fistulous communication that connected separately with both the left and right anterior portal veins. The ductus venosus was occluded.

There was a marked calibre change of the abdominal aorta immediately distal to the IAPF. The distal abdominal aorta and the distal arterial branches were uniformly hypoplastic. The patient had an unusual variant of coeliac axis anatomy, with the hepatic artery arising as a separate vessel from the aorta. The other branches all arose from the remaining gastro-splenic trunk.

Angiography was undertaken in order to embolize the cIAPF. Access was obtained by direct transhepatic puncture of the fistula under ultrasound and fluoroscopic guidance. Aortography and selective angiography were then performed using a 4-French multipurpose catheter inserted through a 4-French sheath in the fistula.

**Figure 3.**
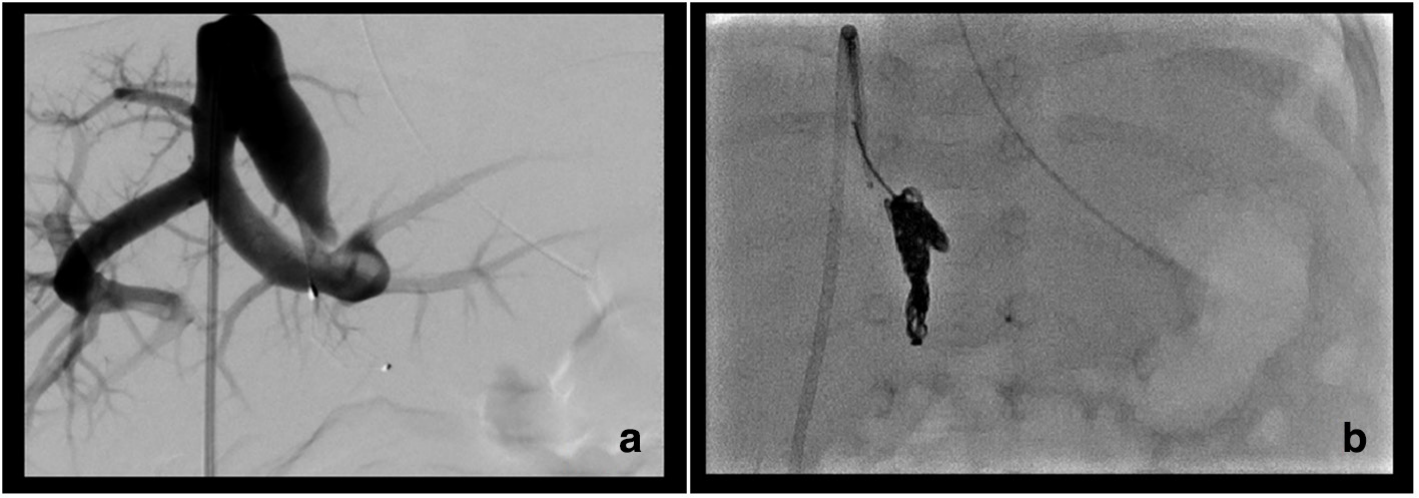
First embolization showing the patent fistula (a) and successful occlusion at the end of the procedure (b).

Angiography delineated the anatomy of the fistula, which was then occluded using a microvascular plug (MVP-7; Medtronic Limited, Watford, UK) placed within the proximal, arterial component of the fistula. A 60 cm packing coil (POD packing coil; Penumbra Europe GmbH, Berlin, Germany) was inserted immediately behind the device to provide additional support (Figure 3a and b).

Angiography confirmed complete occlusion of the cIAPF following insertion of the device. This was accompanied by an immediate intraoperative normalization of the heart rate. Repeat ultrasound on the day after embolization confirmed complete occlusion of the shunt and restoration of normal hepatopetal flow in the portal venous system. The hepatic artery was patent with a normalized arterial flow pattern.

The child recovered well from his procedure and showed clinical improvement from the first day post-procedure. He became more active and began to feed well. His NT pro-BNP showed an immediate and rapid decline. It reduced to 636 ng ml^−1^ the next day and normalized within two weeks following embolization.

A follow-up ultrasound at 3 months after the embolization revealed successful shunt occlusion and the child continued to thrive and gain weight. Subsequent follow-ups were uneventful on clinical and ultrasound examinations until 1 year after the embolization.

At this point in time, the presentation was with recurrent respiratory illness associated with a refusal to gain weight and a poor appetite. These were investigated and a repeat NT-pro-BNP level was performed. This was found to be significantly elevated (939 ng ml^−1^). An ultrasound at this time revealed recurrent arterio-portal shunting. This was investigated further with an angiogram. The child was taken up for the procedure with a view to perform embolization as necessary/technically feasible.

**Figure 4.**
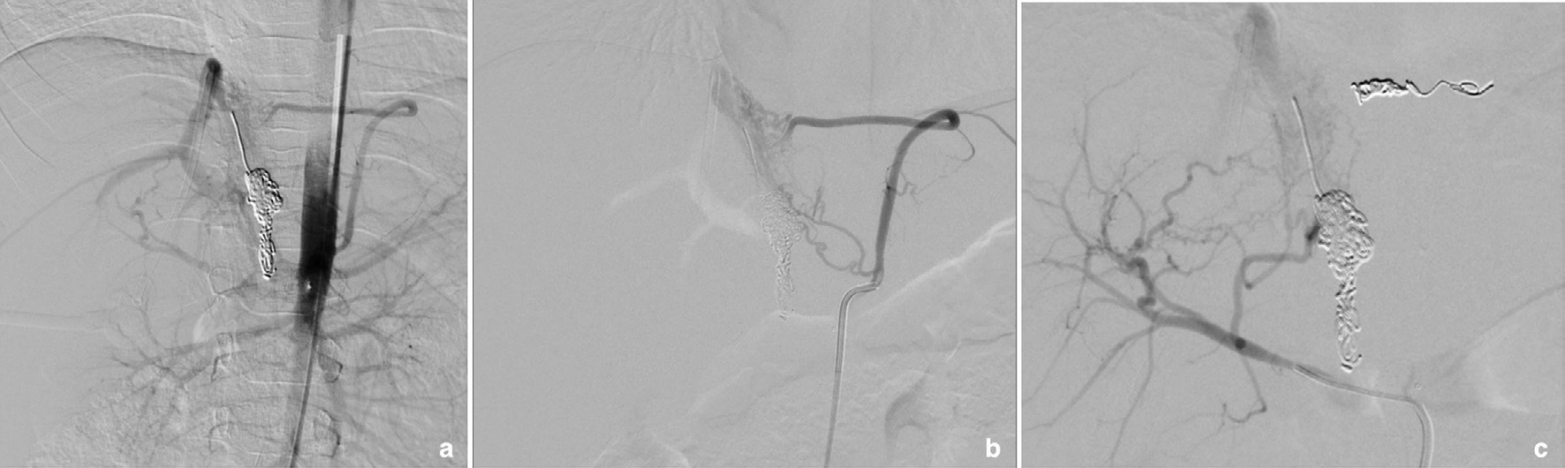
Selective angiography revealing recurrent fistulae above and below the coeliac axis.

The angiogram revealed recurrent arterio-portal shunts. There were two main feeders: one above (left-sided with respect to the aorta) and one below (right-sided) the coeliac axis. Portal angiography revealed a large patent fistula (Figure 4a, b and c).

The left-sided arterial branch was embolized with three 3 m×14 cm micro Nestor coils and a 4 mm×6 cm Ruby coil. Further branches from the right hepatic artery (arising from the aorta) and segmental branches from the segment four artery were also embolized.

An additional vessel was noted from the right adrenal artery and this was also coiled with a 4 mm×6 cm Ruby coil.

**Figure 5.**
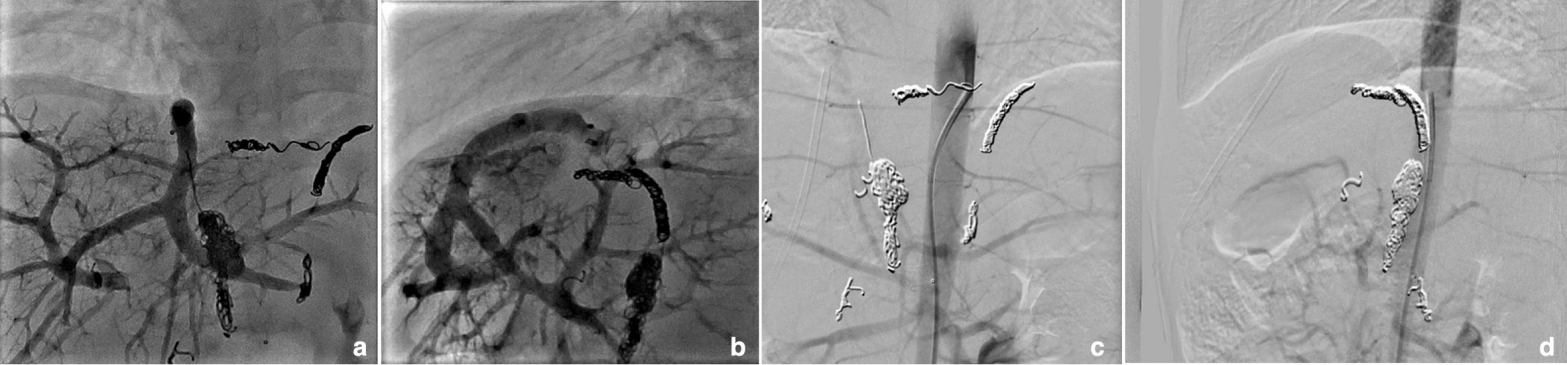
Post-embolization images of the recurrent fistulae showing good occlusion on selective portal angiography (a) and the final position of coils (b).

The final angiogram showed excellent occlusion with no residual shunting (Figure 5a and b). The child was recovered from the procedure uneventfully and experienced one episode of fever after the procedure on the second post-procedure day. This was managed conservatively, and the child was discharged home.

Clinical improvement was again seen from the first day after the procedure. The first notable change was in his feeding and also the resolution of the respiratory symptoms. On further clinical follow-up, 3 and 6 months from the procedure, the child was noted to have gained back all the lost weight with good catch-up growth. Subsequent ultrasound scans have revealed normal flow in the liver vasculature with no evident shunts. The NT pro-BNP levels have dropped significantly since the second procedure, with the latest known level on follow-up being 93 ng ml^−1^ (upper limit of normal- 85 ng ml^−1^).

## Discussion

Congenital IAPF are rare, high-flow hepatic vascular malformations. While the overall incidence remains unknown, only 42 cases have been described that meet the “Norton-Jacobson” classification^2^ since the original report in 1967.^4^

In most patients, cIAPF increase portal venous blood flow and cause presinusoidal portal hypertension with its associated adverse sequelae.^5^ The severity of symptoms vary according to the site of the IAPF and the degree of shunting.^3,5^ Children usually present within the first 3 years, most commonly with upper gastrointestinal bleeding, failure to thrive and chronic diarrhoea or steatorrhoea.^2^ Presentation may be delayed, especially in patients where the ductus venosus remains patent.^6^

Patients with cIAPF rarely develop congestive heart failure or pulmonary hypertension.^3^ This is in stark contrast to patients with systemic arteriovenous shunts. It is hypothesized that the hepatic sinusoids impose a significant resistance to blood flow through the liver.^3^ In those cases where patients present with cIAPF and heart failure, the heart failure has almost always been attributed to concomitant congenital heart disease,^7^ hepatic haemangiomata^4^ or the presence of a patent ductus venosus that allows high-flow shunting between the systemic arterial and systemic venous systems.^2^

As this case clearly highlights, however, cIAPF may be associated with the development of congestive cardiac failure even when the ductus venosus is closed. To date, this is only the second child to have presented with cIAPF and cardiac failure. Vauthey et al reported a 2-year-old child with a cIAPF and heart failure with orthopnoea, pedal oedema and hepatomegaly. The child was treated with five embolization procedures over 13 months. Symptoms resolved once the cIAPF was eventually occluded.^3^ In the current case, the clinical improvement was mirrored by a biochemical improvement in the NT pro-BNP level. NT pro-BNP is a clinically established biomarker of heart failure, which provides prognostic information at the time of diagnosis and during follow-up assessments.^8^ It remains unclear what distinguishes these two children from the other children, who presented with symptoms of portal hypertension.

To date, there have been no cases of spontaneous closure of cIAPF. Moreover, cIAPF represent one of the treatable causes of portal hypertension, and treatment is recommended as soon as the lesion is diagnosed.^1–3^ Radiological intervention is the treatment of choice for non-complex cIAPF.^2,3^ By contrast, complex cIAPF are prone to collateralization and recurrence after intervention and may require a combination of surgery and embolization to treat successfully.^2^ In the current case, micro Nestor coils were used for embolization. Gelatine sponge embolization has been also known as an alternative to coil embolization in this setting. The current case was complex (Type 3) by virtue of the fact that the fistula arose directly from the aorta. However, the fistula had a long, single channel that eventually supplied the left and right portal veins. This anatomy made it well-suited to radiological embolization using a direct, transhepatic approach. This approach has not previously been described in the management of cIAPF. It provided excellent, minimally invasive access to the fistula while avoiding the need for either transfemoral arterial or transhepatic portal venous access, with their associated risks.^2,3,5^

The long-term prognosis for children with cIAPF following treatment remains unknown.^2^ Most children become asymptomatic shortly after treatment and show excellent catch up growth.^4^ However, in cases with more severe arterialization of the portal venous system or multiple intrahepatic fistulae, some children have required liver transplantation in order to manage their persistent or recurrent portal hypertension.^4^

While a recurrent fistula was unexpected, we do expect that this child should have an excellent long-term outcome, given that there was no evidence of established portal hypertension at presentation or at the recurrence. Follow-up includes routine clinical evaluation to ensure good catch up growth and serial ultrasounds to evaluate the status of the occluded fistula. This strategy has, in this case, proven to successfully pick up any recurrences.

## Learning points

- Congenital intrahepatic arterio-portal fistulae are rare and can present atypically (in this case with cardiac failure).
- Angiographic embolization is effective with resolution of symptoms in cases where it is technically feasible.
- Serial clinical monitoring (in addition to N-terminal pro-brain natriuretic peptide in this case) is essential to diagnose recurrent fistulae.

## Conclusions

Congenital IAPF represent one of the treatable causes of portal hypertension. However, as this case illustrates, they may also rarely present with heart failure. The symptoms of heart failure resolve following successful and complete embolization of the shunt. This case also affirms the role of interventional radiology in the management of even complex cIAPF, by occluding the shunt and avoiding the complications of major abdominal surgery.
